# Supplementary material for: Effect of Activator and Outgoing Ligand Nature on the Catalytic Behavior of Bis(phenoxy-imine) Ti(IV) Complexes in the Polymerization of Ethylene and Its Copolymerization with Higher Olefins
Source: Polymers (Basel). 2022 Oct 18;14(20):4397. doi: 10.3390/polym14204397 (PMC9609217; doi:10.3390/polym14204397)
Supplement: Supplementary file 1 [file polymers-14-04397-s001.zip › polymers-1956520-supplementary v2 rev-highlight.pdf]

SUPPORTING INFORMATION

Effect of activator and outgoing ligand nature on the catalytic behavior of bis(phenoxy-imine) Ti(IV) complexes in the polymerization of ethylene and its copolymerization with higher olefins

Svetlana Ch. Gagieva, Kasim F. Magomedov, Vladislav A. Tuskaev, Vyacheslav S. Bogdanov, Dmitrii A. Kurmaev, Evgenii K. Golubev, Gleb L. Denisov, Galina G. Nikiforova, Maria D. Evseeva, Daniele Saracheno, Mikhail I. Buzin, Pavel B. Dzhevakov, Viktor I. Privalov, Boris M. Bulychev

Table S1. Crystal data and structure refinement parameters for **$(L1)_2Ti(OiPr)_2$**, **$(L1)_2Zr(OiPr)_2$**, **$(L2)_2Zr(OiPr)_2$**, and **$(L1)_2Zr(Cl)_2$**.

Parameter	$(L1)_2Ti(OiPr)_2$	$(L1)_2Zr(OiPr)_2$	$(L2)_2Zr(OiPr)_2$	$(L1)_2Zr(Cl)_2$
Formula unit	$C_{48}H_{66}N_2O_4Ti$	$C_{48}H_{66}N_2O_4Zr$	$C_{48}H_{58}F_8N_2O_4Zr$	$C_{42}H_{52}Cl_2N_2O_2Zr$
Formula weight	782.92	826.24	970.18	822.06
Temperature, K	100	100	100	100
Crystal system	Orthorhombic	Monoclinic	Triclinic	Monoclinic
Space group	$P2_12_12_1$	$P2_1/c$	P-1	$P2_1/c$
Z	4	4	4	4
a, Å	11.5070(6)	9.1474(3)	16.8117(5)	16.2094(3)
b, Å	14.6000(10)	21.9741(7)	16.9626(5)	14.8601(3)
c, Å	26.8041(18)	22.5945(7)	17.6900(5)	18.0347(3)
α, °	90	90	93.357(2)	90
β, °	90	92.955(2)	108.4060(10)	96.1680(10)
γ, °	90	90	94.947(2)	90
V, $Å^3$	4503.1(5)	4535.6(3)	4749.1(2)	4318.93(14)
D_{calc} (g cm^{-3})	1.155	1.210	1.357	1.264
Linear absorption, μ (cm^{-1})	2.33	2.85	3.07	4.15
F(000)	1688	1760	2016	1732
$2\Theta_{max}$, °	54	54	56	52
Reflections measured	52677	49056	58184	44108
Independent reflections	10881	9908	22897	8473
Observed reflections [$I > 2\sigma(I)$]	9596	8599	19314	7876
Parameters	516	524	1167	486
R1	0.0408	0.0986	0.0425	0.0307
wR2	0.0937	0.1991	0.1080	0.0807
GOF	1.037	1.379	1.021	1.047
$\Delta\rho_{max}/\Delta\rho_{min}$ (e $Å^{-3}$)	0.449 / -0.266	1.803 / -1.099	1.182 / -1.151	1.125 / -0.686

Table S2. Selected bond lengths (A) and angles (deg) for complexes **$(L1)_2Ti(OiPr)_2$, $(L1)_2Zr(OiPr)_2$, $(L2)_2Zr(OiPr)_2$ and $(L1)_2Zr(Cl)_2$.**

	**$(L1)_2Ti(OiPr)_2$**	**$(L1)_2Zr(OiPr)_2$**	**$(L2)_2Zr(OiPr)_2$***	**$(L1)_2Zr(Cl)_2$**
M(1)–O(1)	1.9144(18)	2.039(3)	2.0352(13)	1.9699(12)
M(1)–O(2)	1.8051(19)	1.940(4)	1.9076(13)	-
M(1)–O(1A)	1.9176(18)	2.040(3)	2.0324(13)	1.9893(12)
M(1)–O(2A)	1.7849(19)	1.928(4)	1.9129(14)	-
M(1)–N(1)	2.242(3)	2.400(4)	2.4785(16)	2.3810(15)
M(1)–N(1A)	2.326(2)	2.422(5)	2.4901(16)	2.3573(14)
O(1A)–M(1)–O(1)	161.58(8)	161.04(16)	154.33(5)	165.47(5)
N(1)–M(1)–O(2A)/ N(1)–Zr(1)–Cl(2)	166.94(9)	160.50(18)	162.45(6)	162.97(4)

* The asymmetric unit of **$(L2)_2Zr(OiPr)_2$** contains two independent molecules **A** and **B** close in their geometrical parameters. Selected bond and angles of **B** molecule are presented in table. and.

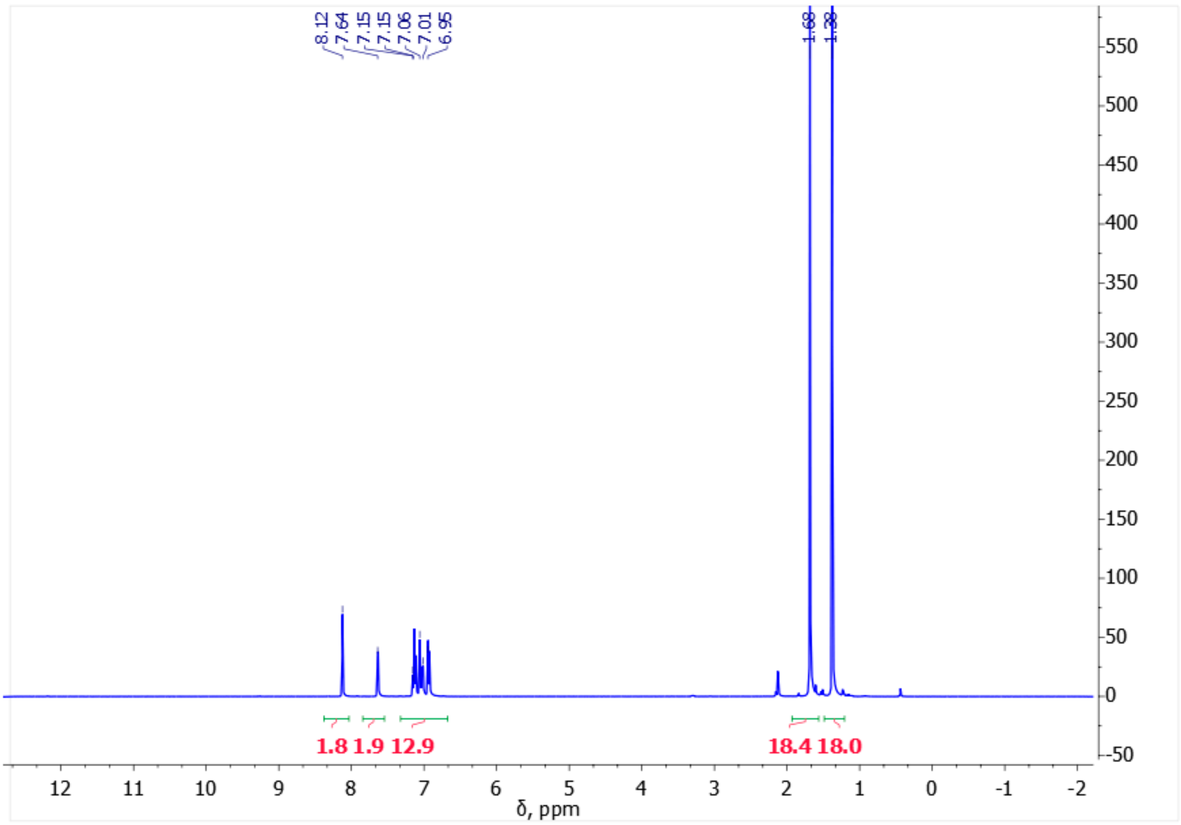

Figure S1. ^{1}H NMR spectrum of **$(L1)_2TiCl_2$**.

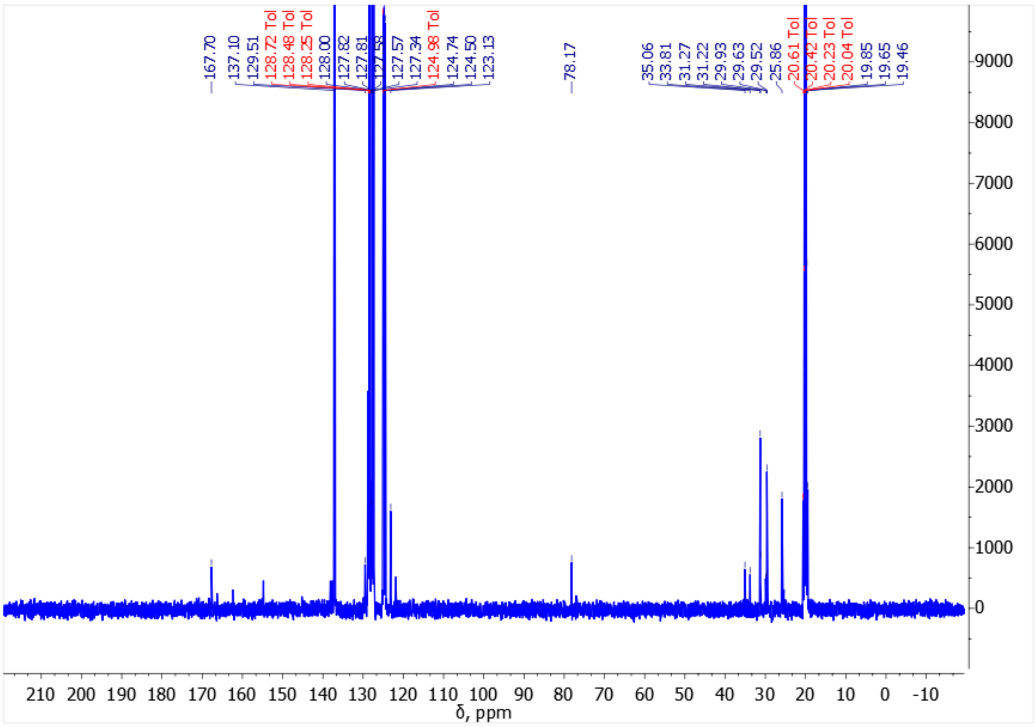

Figure S2. ^{13}C NMR spectrum of **(L1)$_2$TiCl$_2$**.

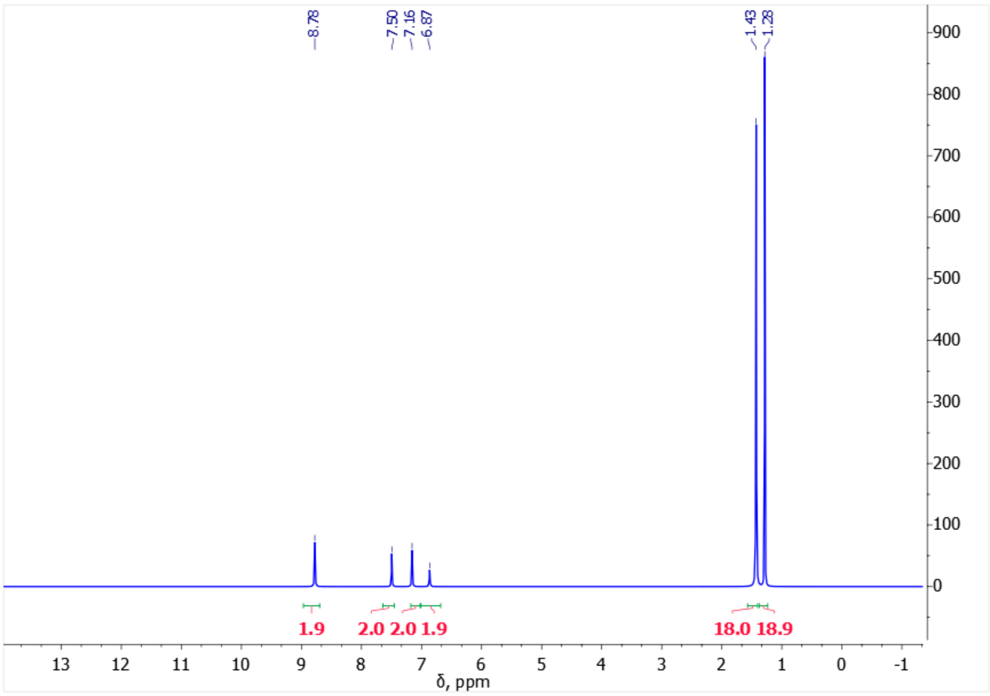

Figure S3. ^{1}H NMR spectrum of **(L2)$_2$TiCl$_2$**.

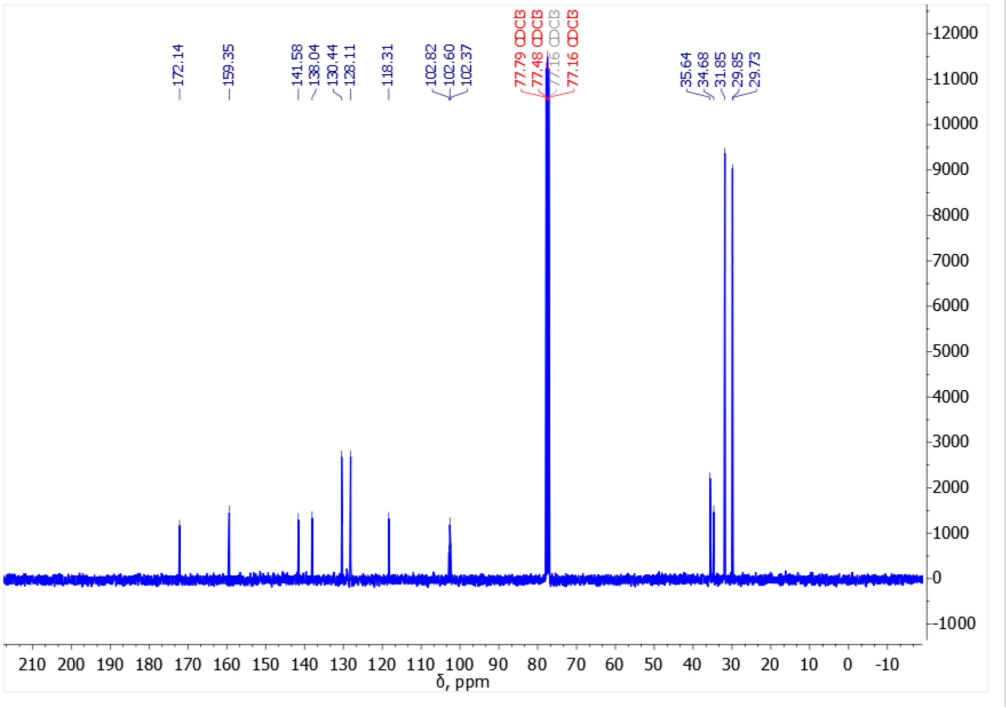

Figure S4. ^{13}C NMR spectrum of **$(L2)_2TiCl_2$**.

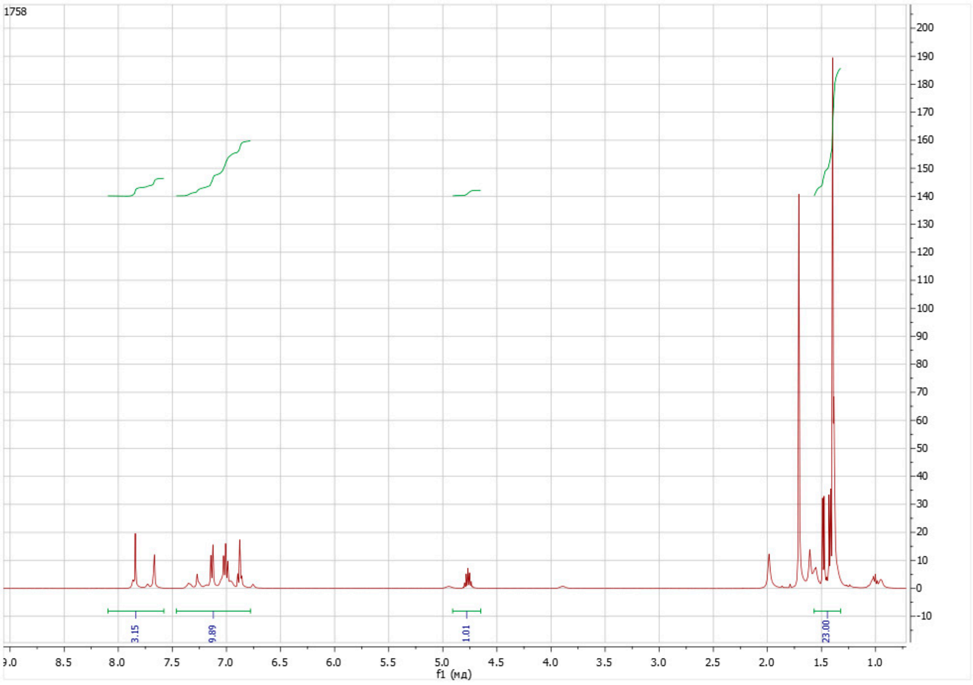

Figure S5. ^{1}H NMR spectrum of $(L1)_2ZrCl_2$.

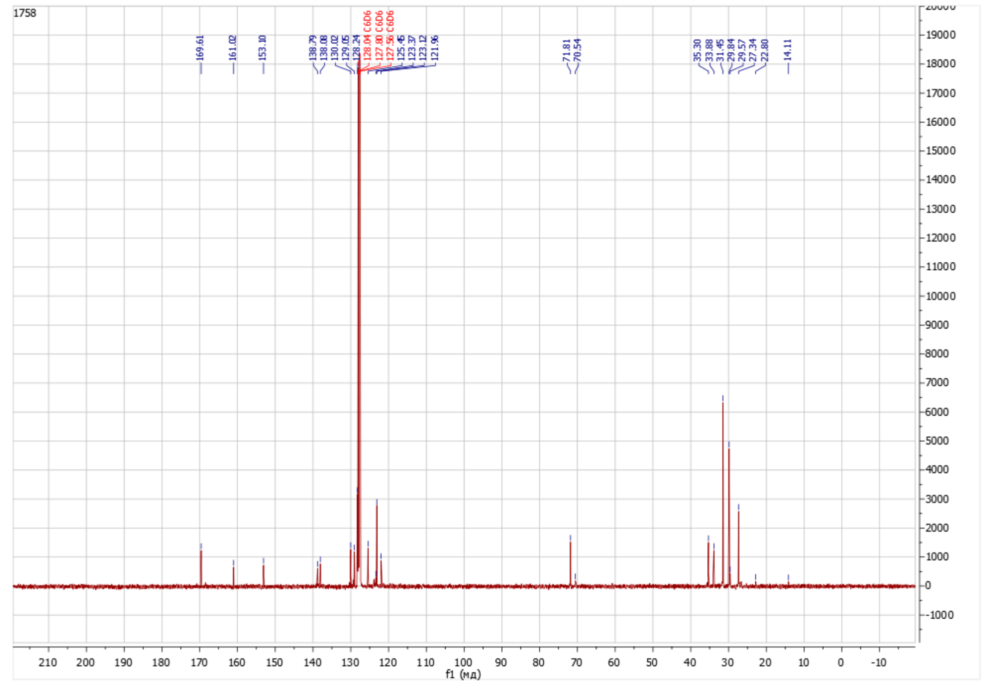

Figure S6. ^{13}C NMR spectrum of $(L1)_2ZrCl_2$.

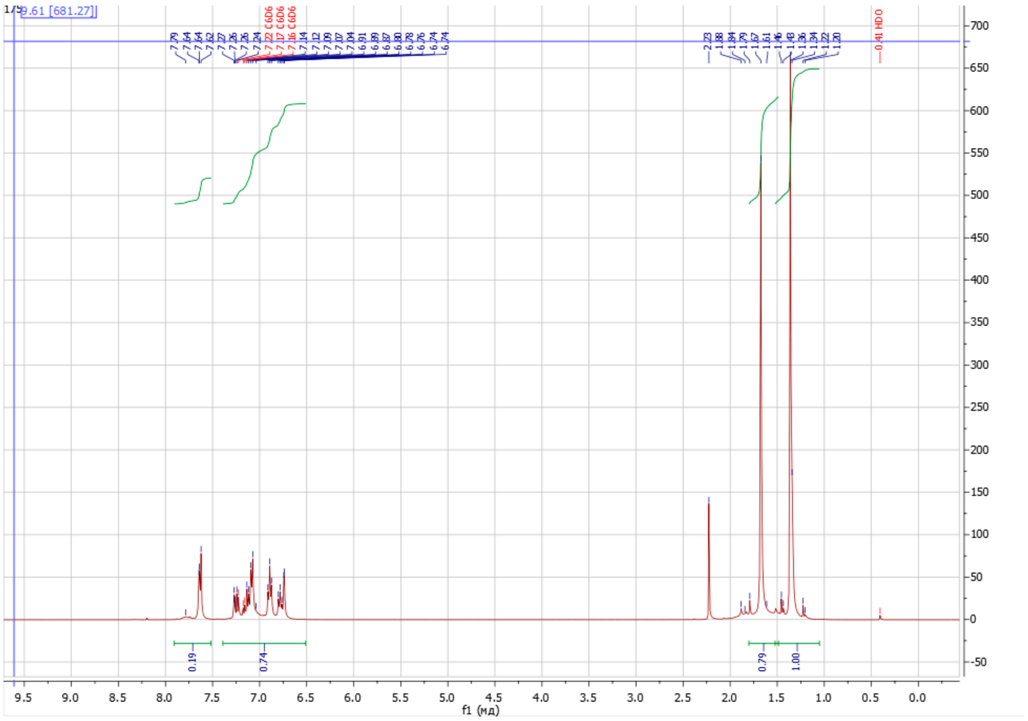

Figure S7. ^{1}H NMR spectrum of **(L1)$_2$**Zr**Cl$_2$**.

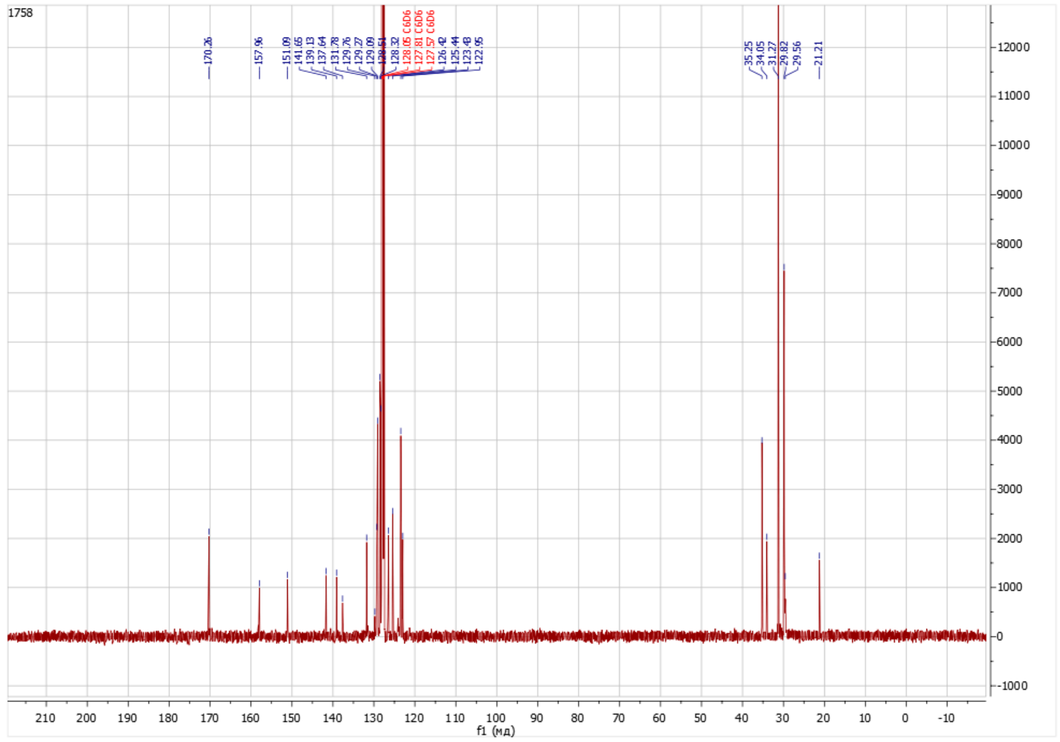

Figure S8. ^{13}C NMR spectrum of $(L1)_2ZrCl_2$.

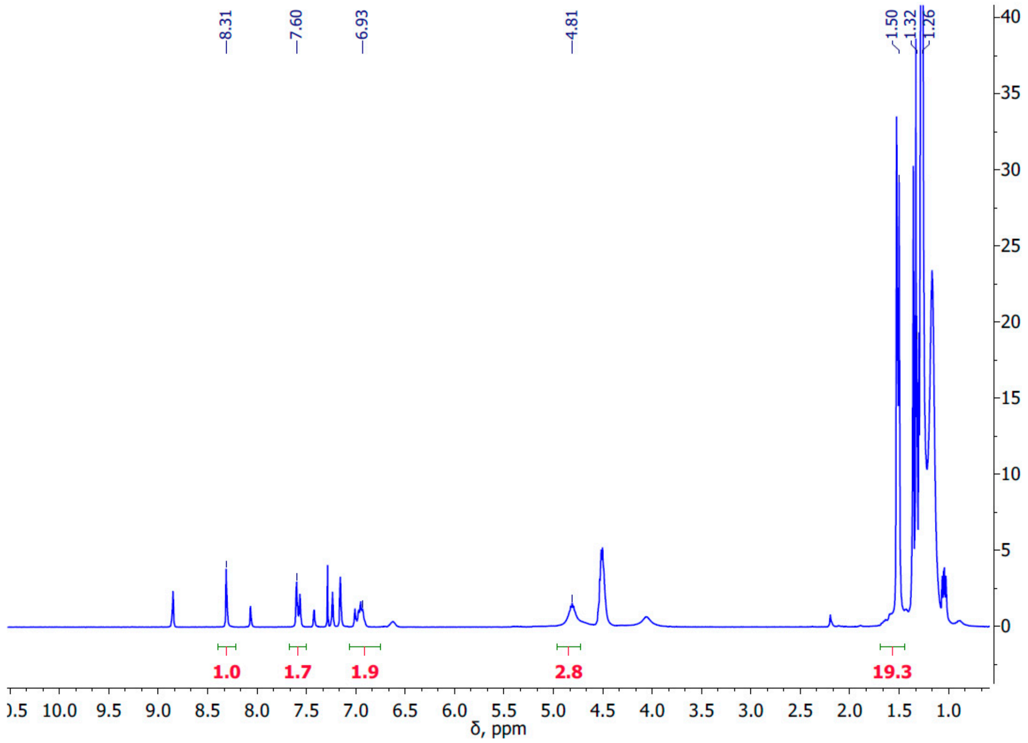

Figure S9. ^{1}H NMR spectrum of $(L1)_2Ti(OiPr)_2$.

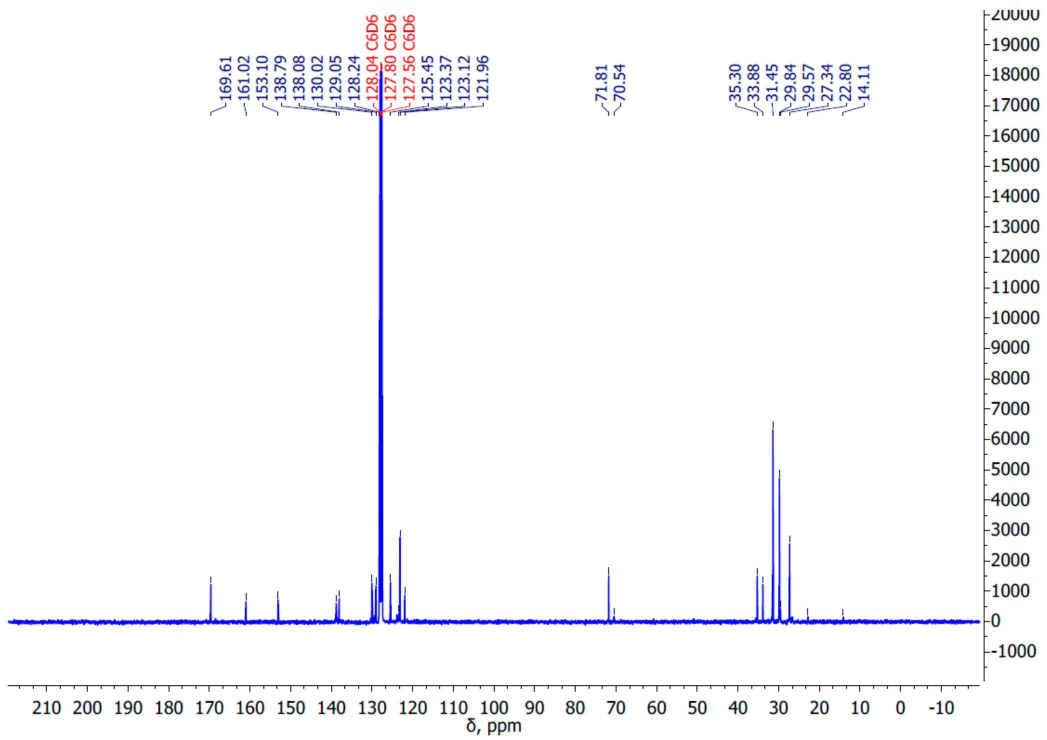

Figure S10. ^{13}C NMR spectrum of $(L1)_2Ti(OiPr)_2$.

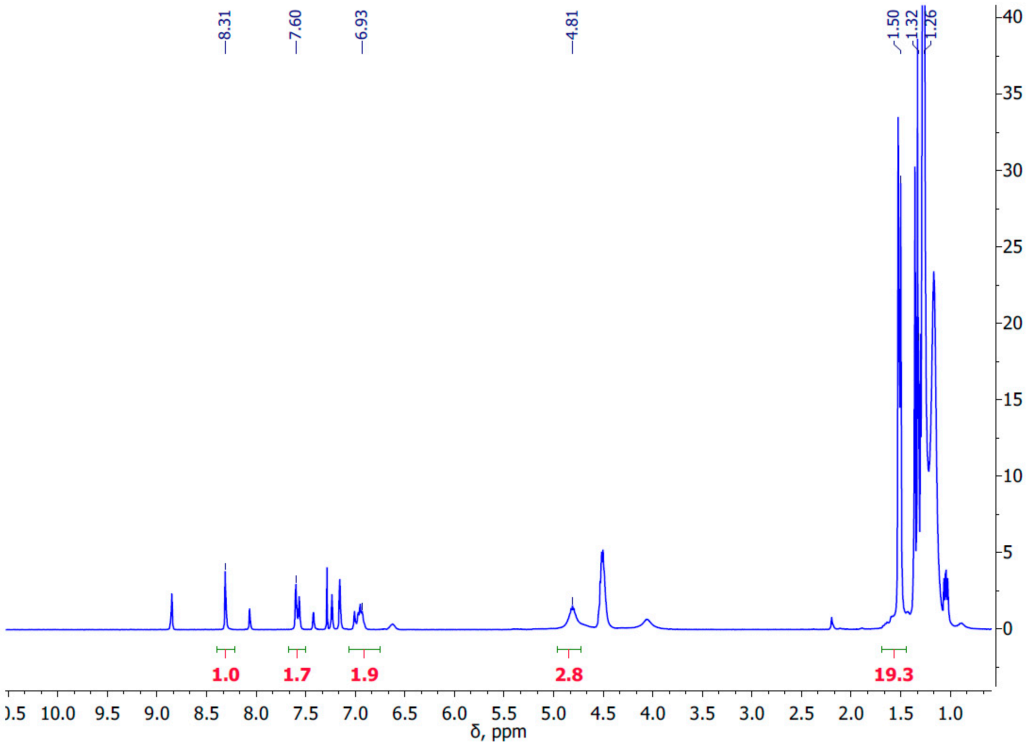

Figure S11. ^{1}H NMR spectrum of $(L2)_2Ti(OiPr)_2$.

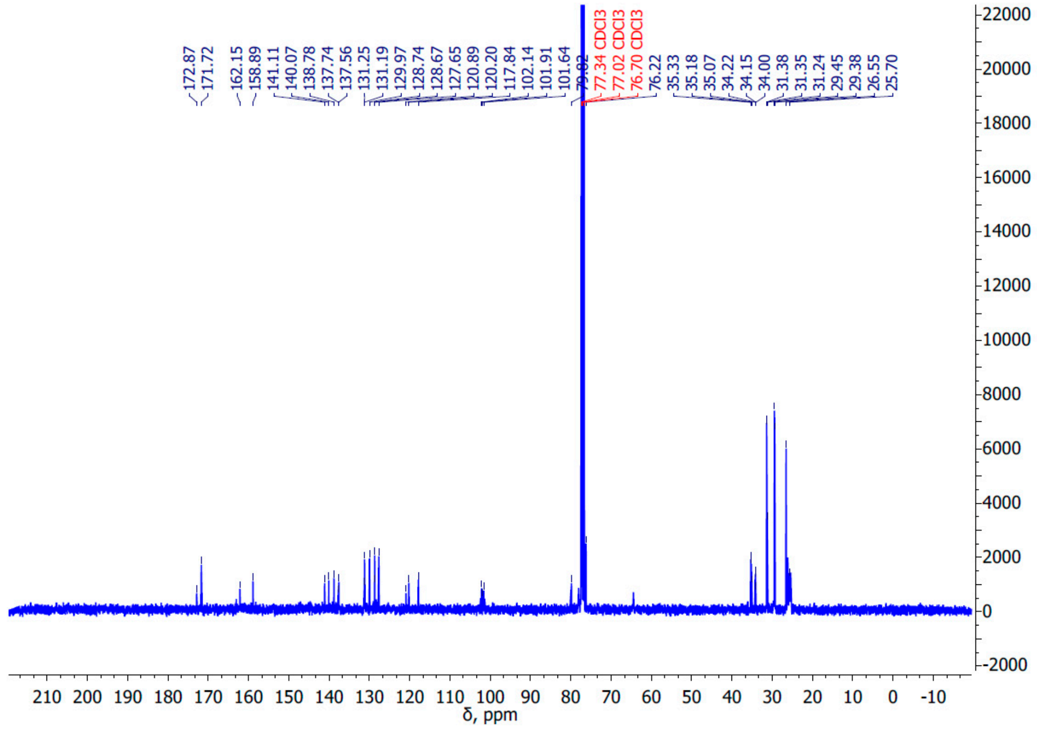

Figure S12. ^{13}C NMR spectrum of $(L2)_2Ti(OiPr)_2$.

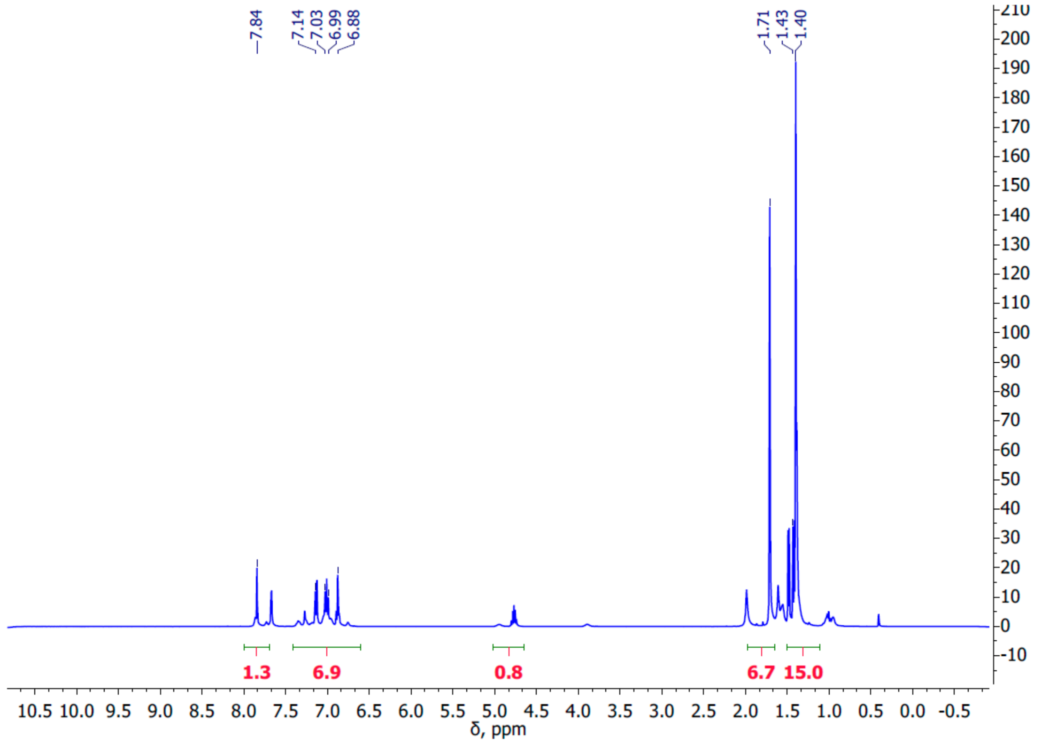

Figure S13. ^{1}H NMR spectrum of $(L1)_2Zr(OiPr)_2$.

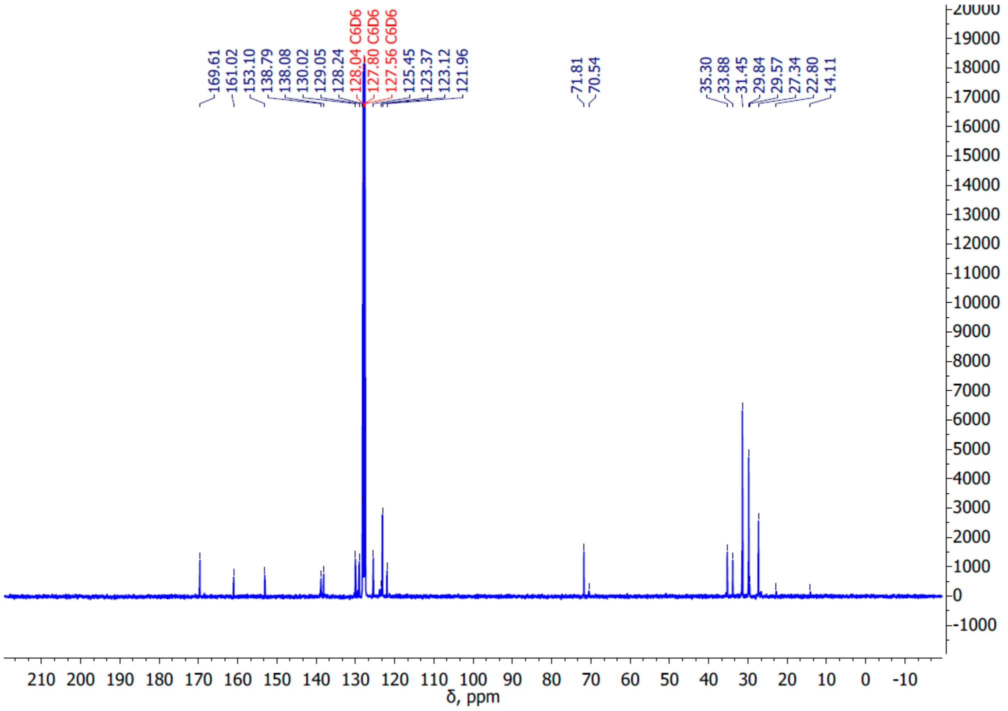

Figure S14. ^{13}C NMR spectrum of $(L2)_2Zr\ (OiPr)_2$.

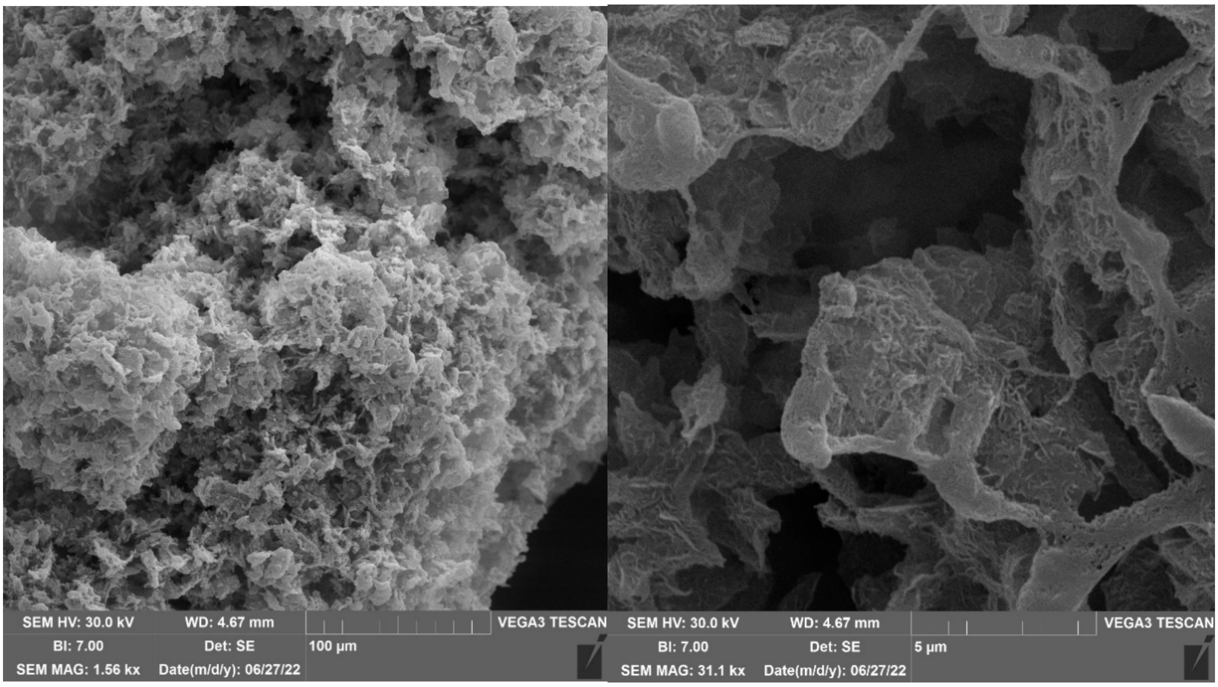

Figure S15. SEM images of the surface morphology of UHMWPE powders obtained with catalytic system **$(L2)_2TiCl_2$/MAO,** 500 eq. (entry 13, Tables 1-2)

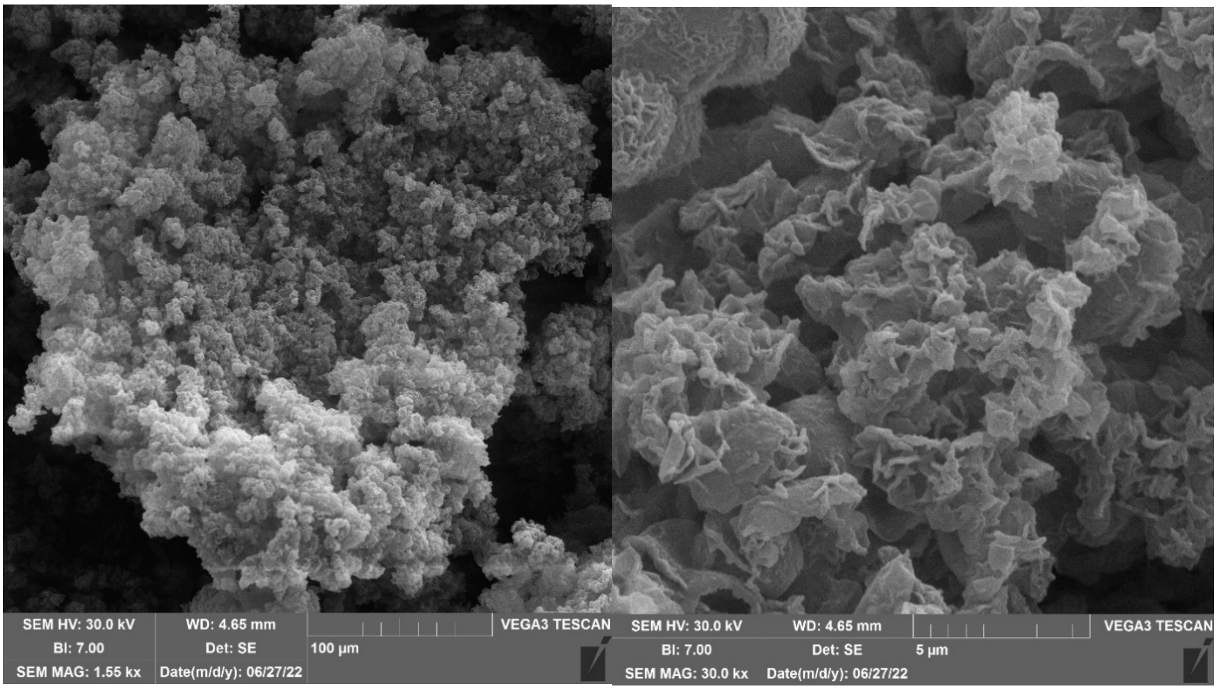

Figure S16. SEM images of the surface morphology of UHMWPE powders obtained with catalytic system **$(L1)_2ZrCl_2$/MAO,** 500 eq. (entry 21, Tables 1-2)

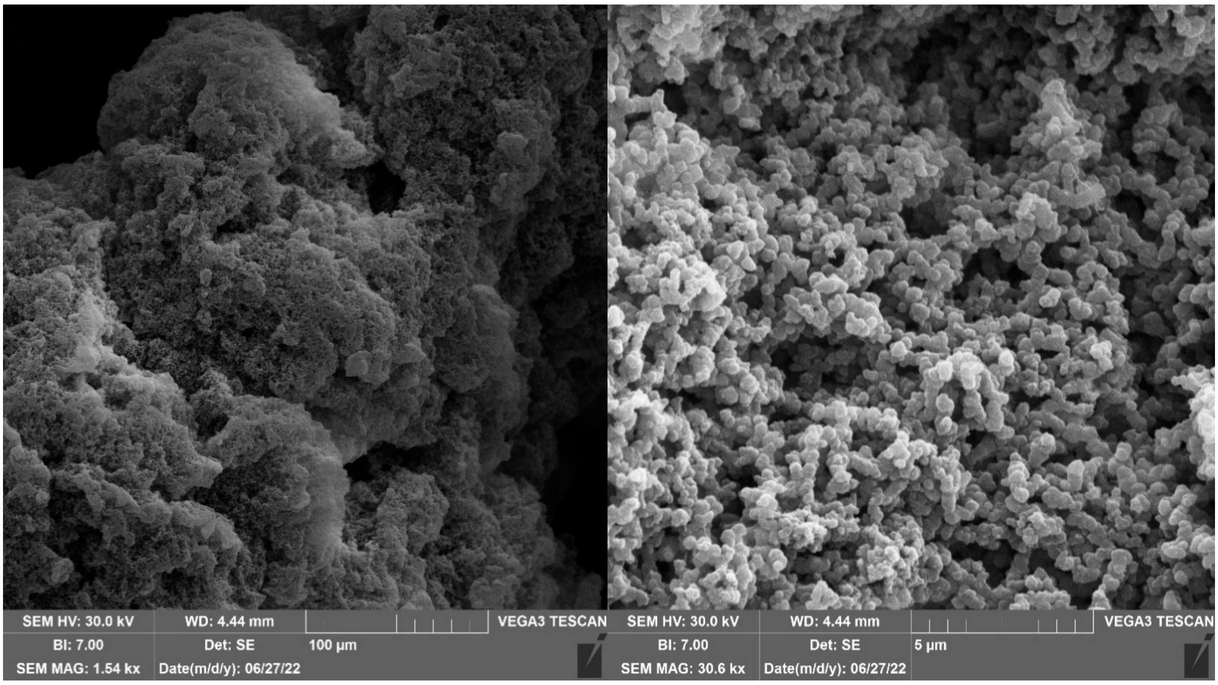

Figure S17. SEM images of the surface morphology of UHMWPE powders obtained with catalytic system **$(L1)_2TiCl_2$**/$Et_3Al_2Cl_3$+Bu_2Mg, (entry 4, Tables 1-2)

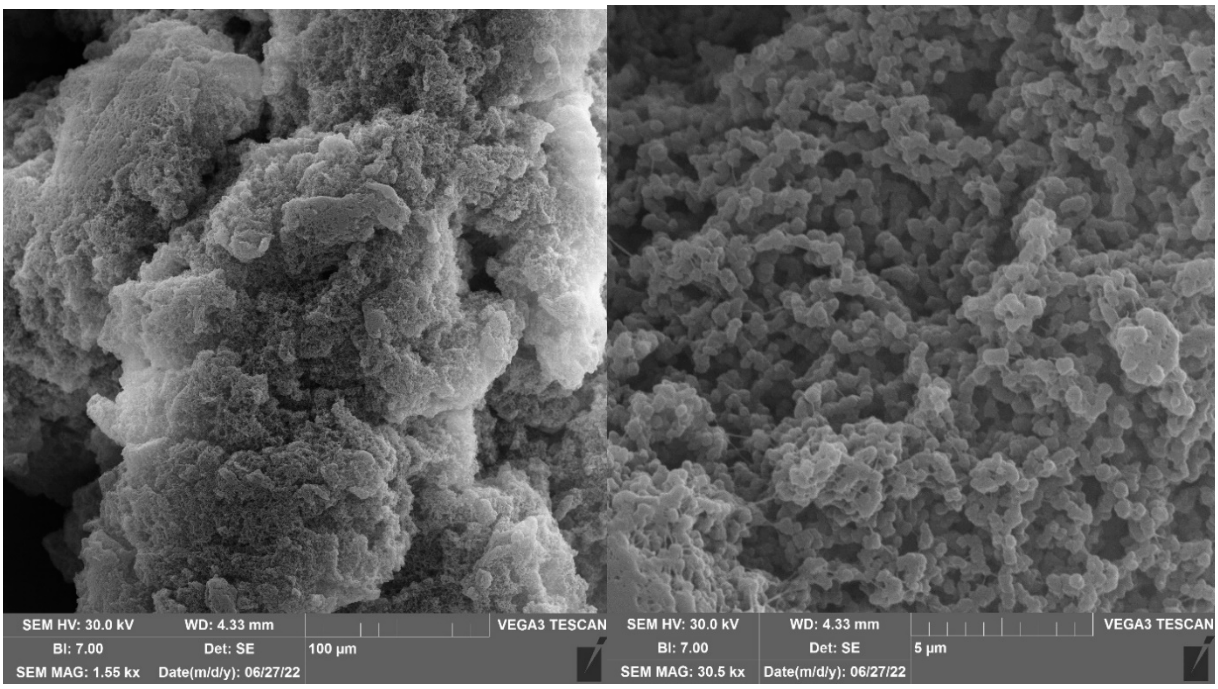

Figure S18. SEM images of the surface morphology of UHMWPE powders obtained with catalytic system **(L1)$_2$Ti(OiPr)$_2$** / Et_2AlCl+Bu_2Mg, (entry 7, Tables 1-2)

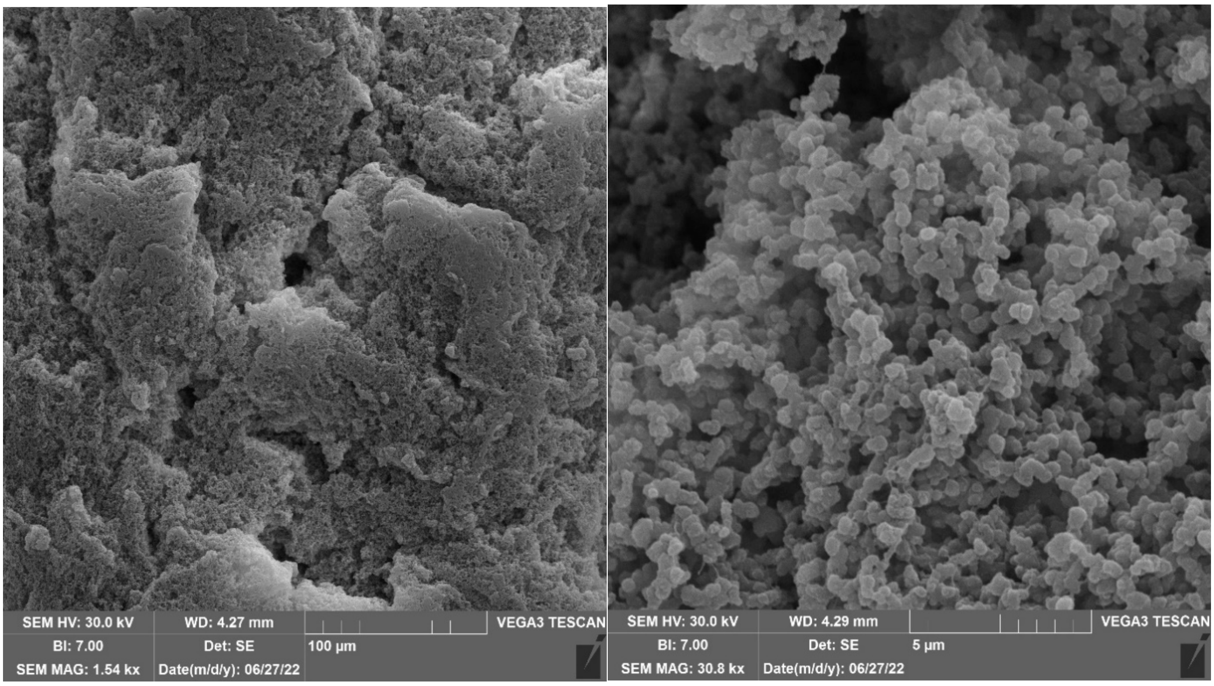

Figure S19. SEM images of the surface morphology of UHMWPE powders obtained with catalytic system **(L1)$_2$Ti(OiPr)$_2$**/$Et_3Al_2Cl_3$+ Bu_2Mg**,** (entry 8, Tables 1-2)

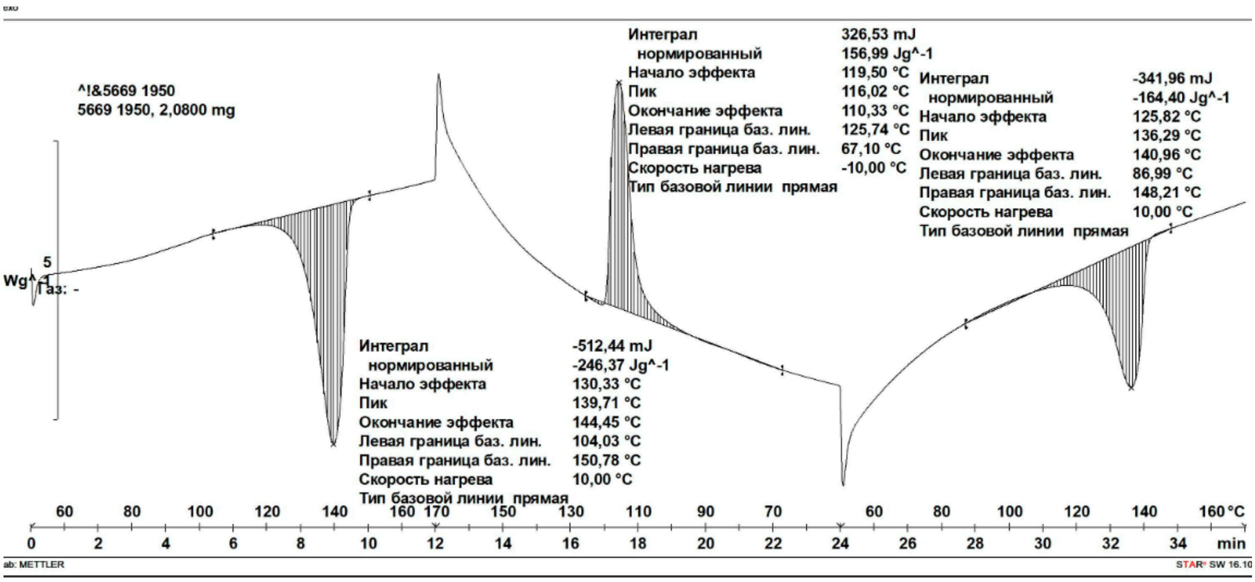

Figure S20. DSC curves corresponding to UHMWPE produced on $(L1)_2TiCl_2/Et_2AlCl/ Bu_2Mg$ =1/300/100 (entry 1, table 1).

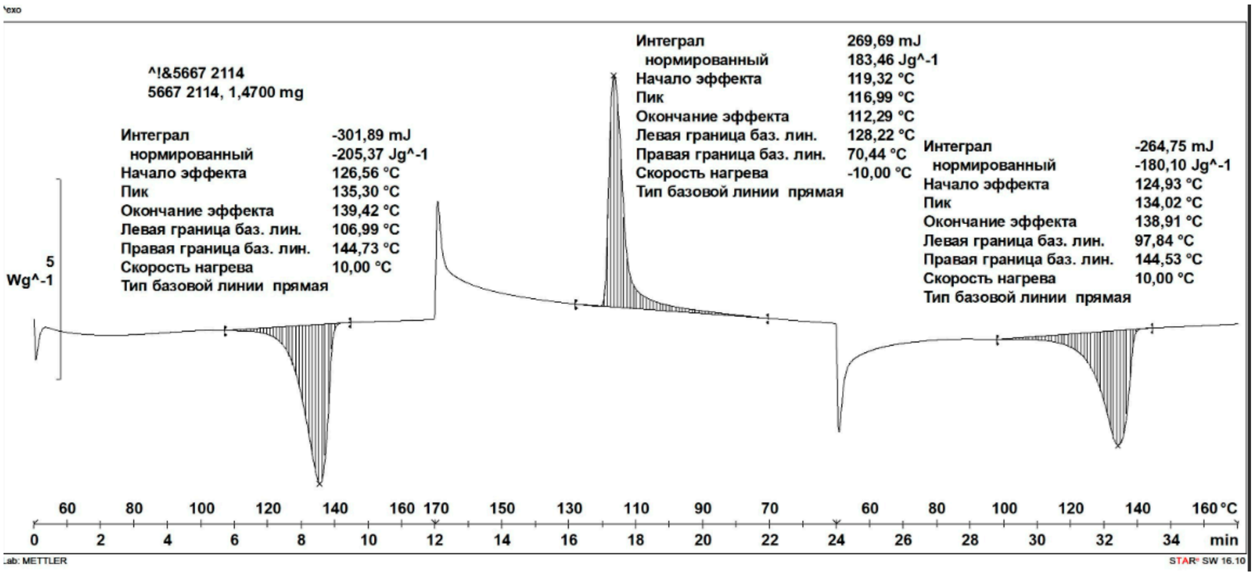

Figure S21. DSC curves corresponding to UHMWPE produced on $(L1)_2TiCl_2/Et_2AlCl/ Bu_2Mg$ =1/300/100 (entry 2, table 1).

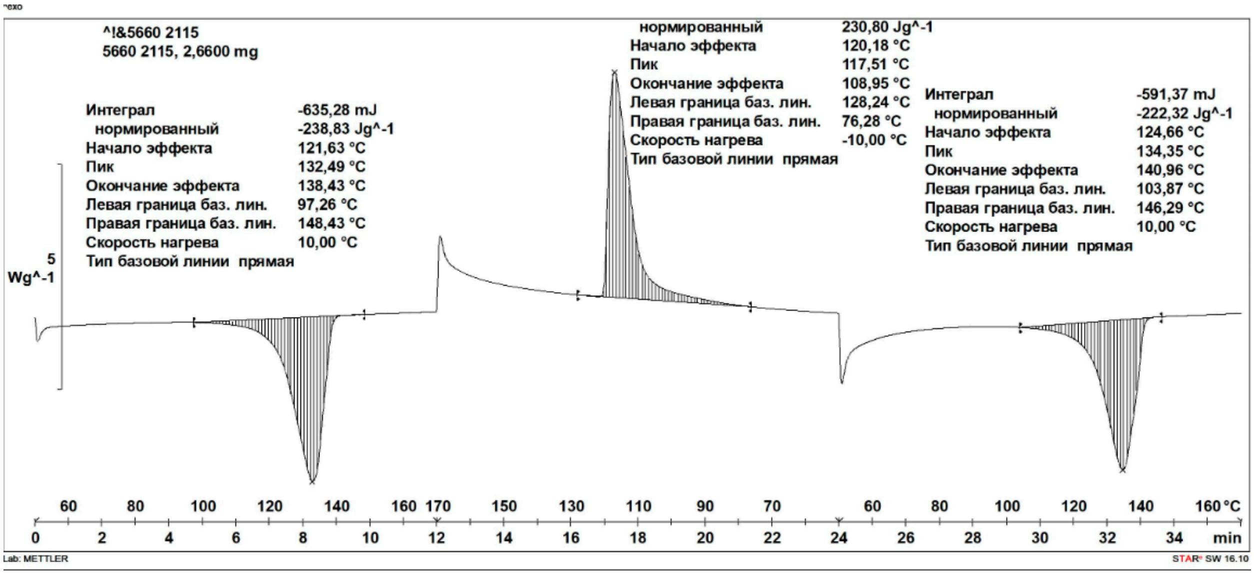

Figure S22. DSC curves corresponding to UHMWPE produced on $(L1)_2TiCl_2/Et_2AlCl/$ Bu_2Mg =1/300/100 (entry 3, table 1).

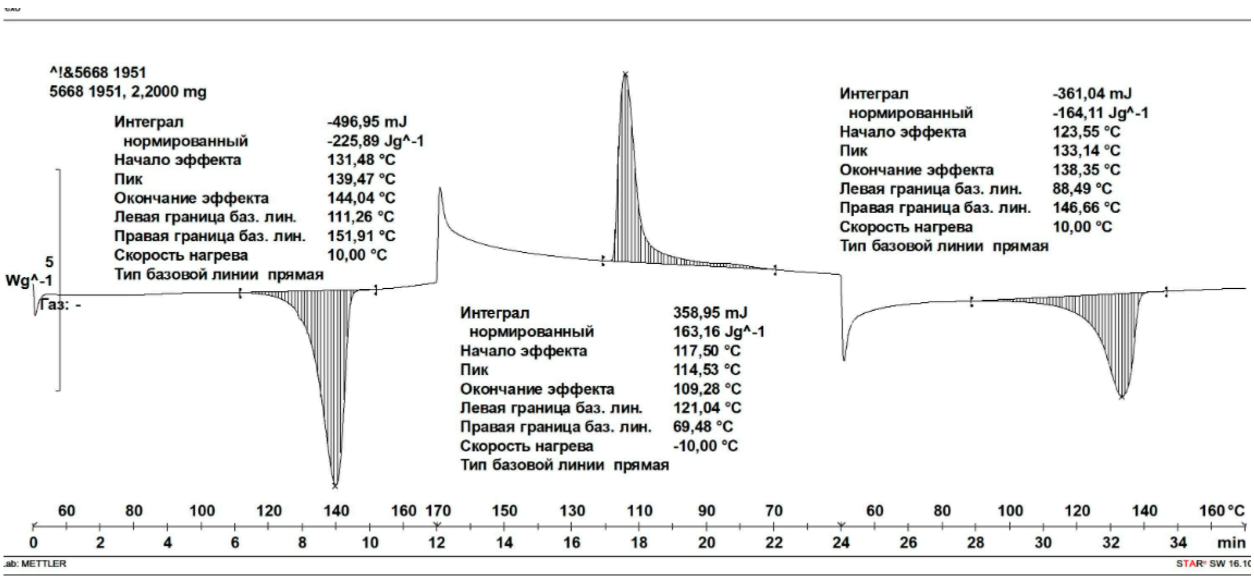

Figure S23. DSC curves corresponding to UHMWPE produced on $(L1)_2TiCl_2$ $/Et_3Al_2Cl_3/$ Bu_2Mg=1/300/100 (entry 4, table 1).

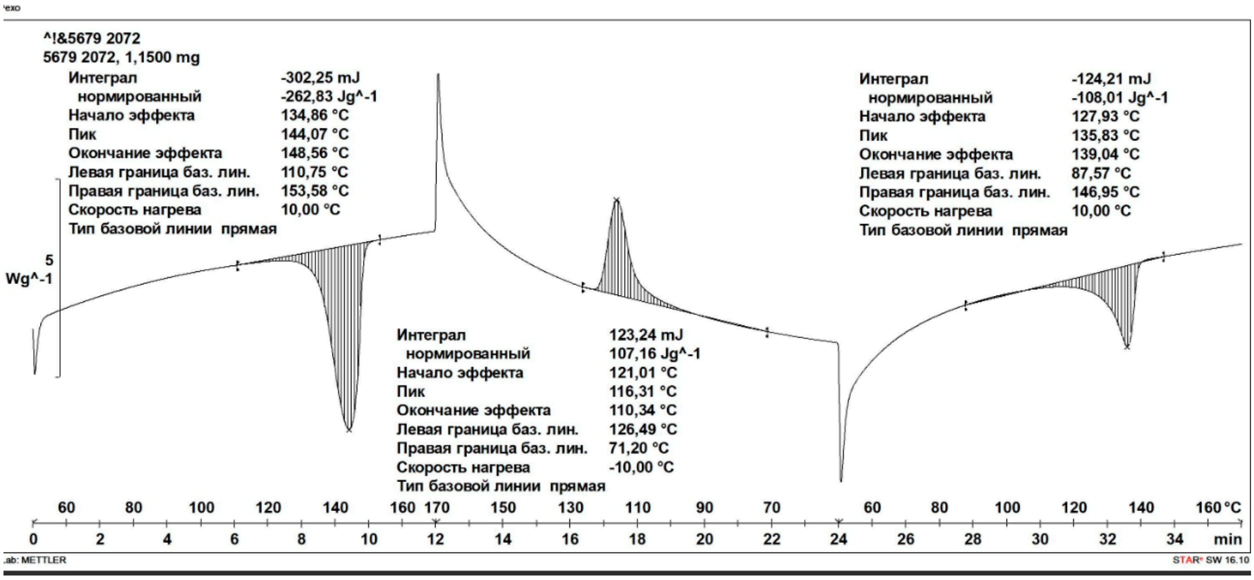

Figure S24. DSC curves corresponding to UHMWPE produced on $(L1)_2TiCl_2$/ MMAO12 = 1/1000 (entry 5, table 1).

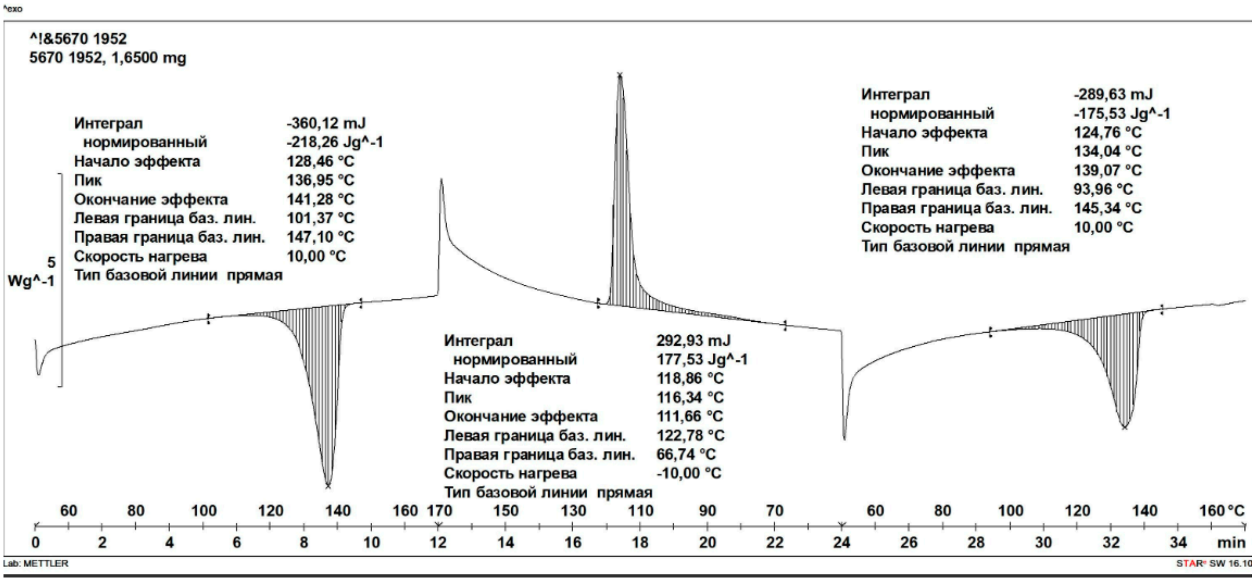

Figure S25. DSC curves corresponding to UHMWPE produced on **$(L1)_2Ti(OiPr)_2$/**Et_2AlCl/Bu_2Mg = 1/300/100 (entry 7, table 1).

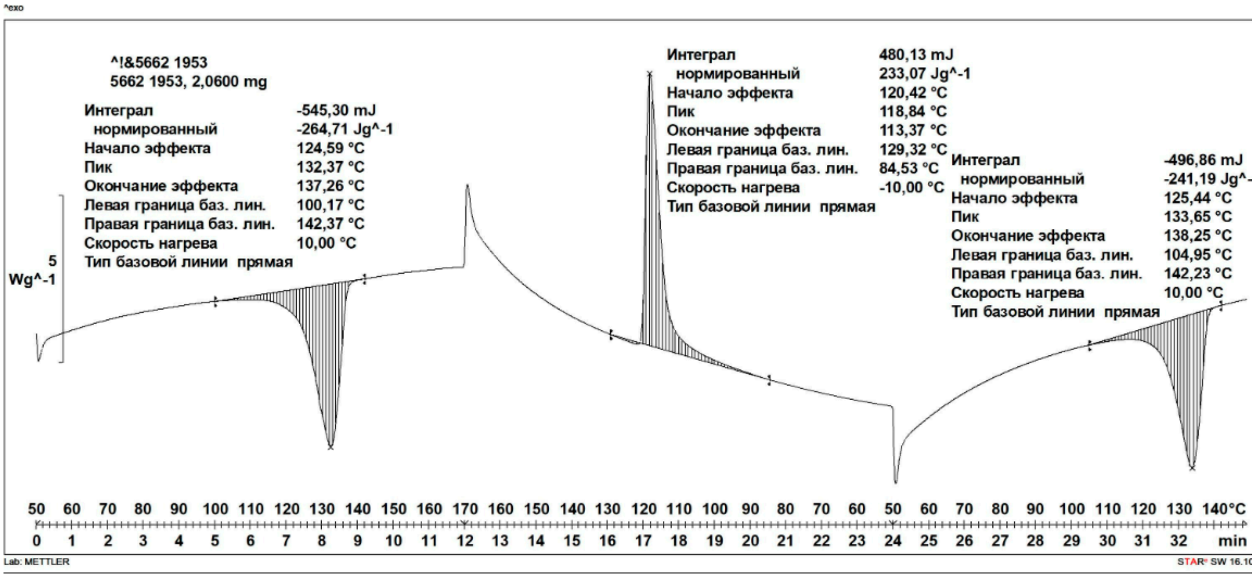

Figure S26. DSC curves corresponding to UHMWPE produced on **(L1)$_2$Ti(OiPr)$_2$/**$Et_3Al_2Cl_3$/Bu_2Mg = 1/300/100 (entry 8, table 1).

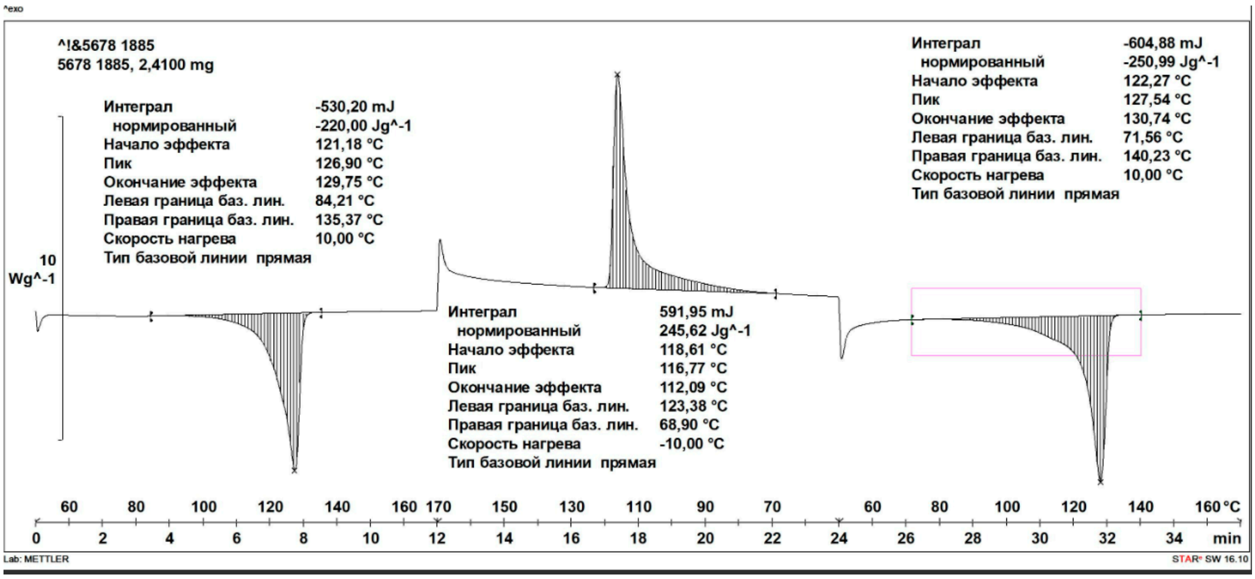

Figure S27. DSC curves corresponding to UHMWPE produced on **(L2)$_2$Ti(OiPr)$_2$/**$Et_3Al_2Cl_3$/Bu_2Mg = 1/300/100 (entry 18, table 1).

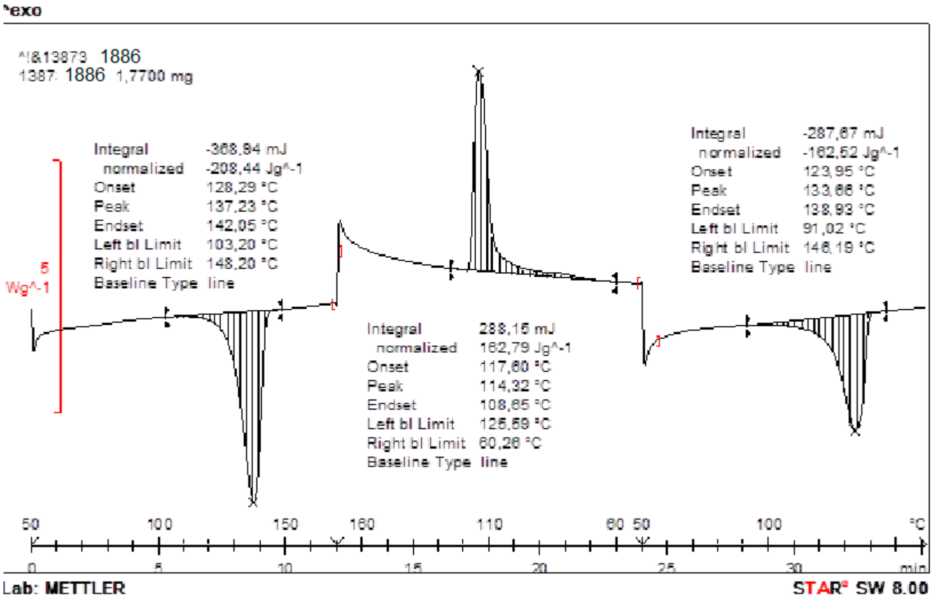

Figure S28. DSC curves corresponding to UHMWPE produced on **$(L2)_2Ti(OiPr)_2$/** MMAO12 = 1/1000 (entry 19, table 1).

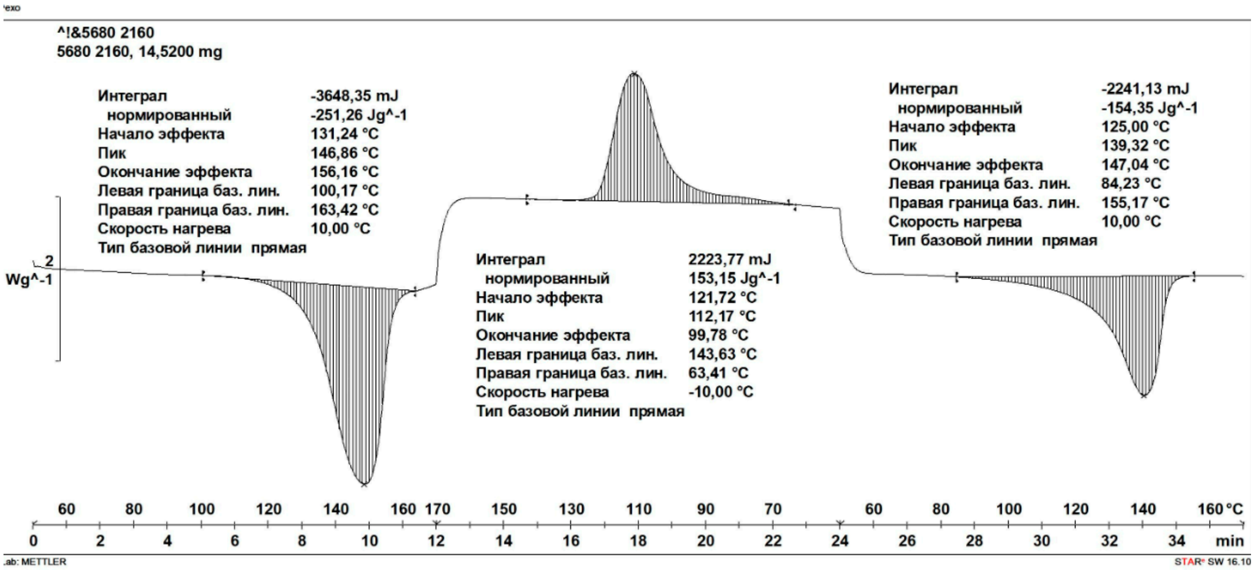

Figure S29. DSC curves corresponding to UHMWPE produced on **$(L1)_2ZrCl_2$**/$(^iBu)_3Al$/ $CPh_3^+B(C_6F_5)_4^-$ = 1/80/2 (entry 20, table 1).

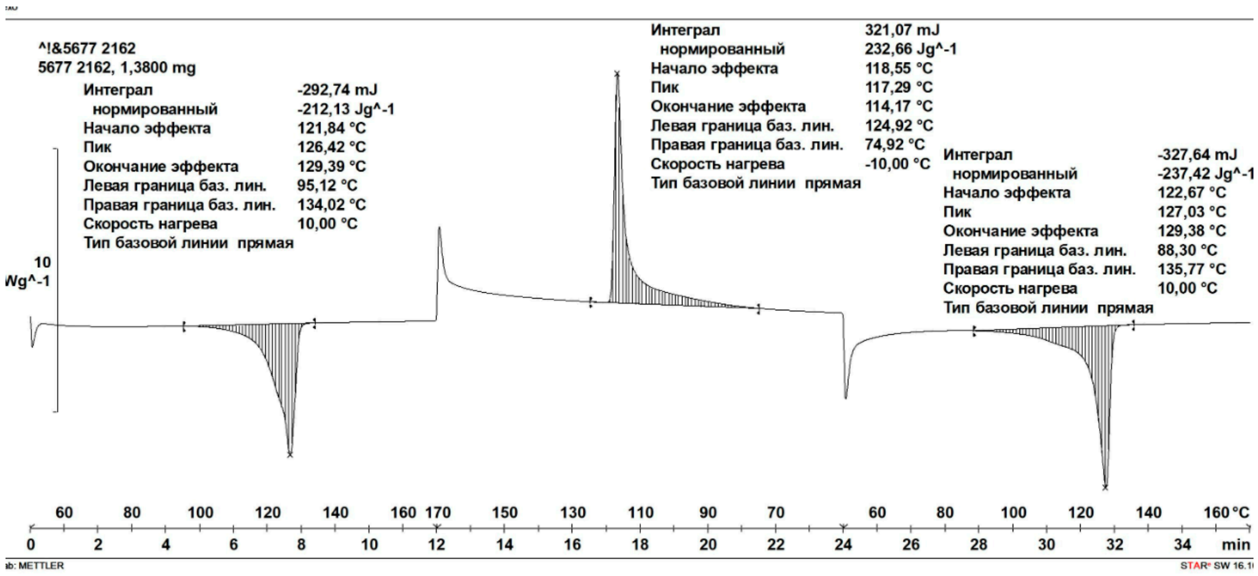

Figure S30. DSC curves corresponding to UHMWPE produced on **(L1)$_2$ZrCl$_2$**/ MMAO12 = 1/500 (entry 21, table 1).

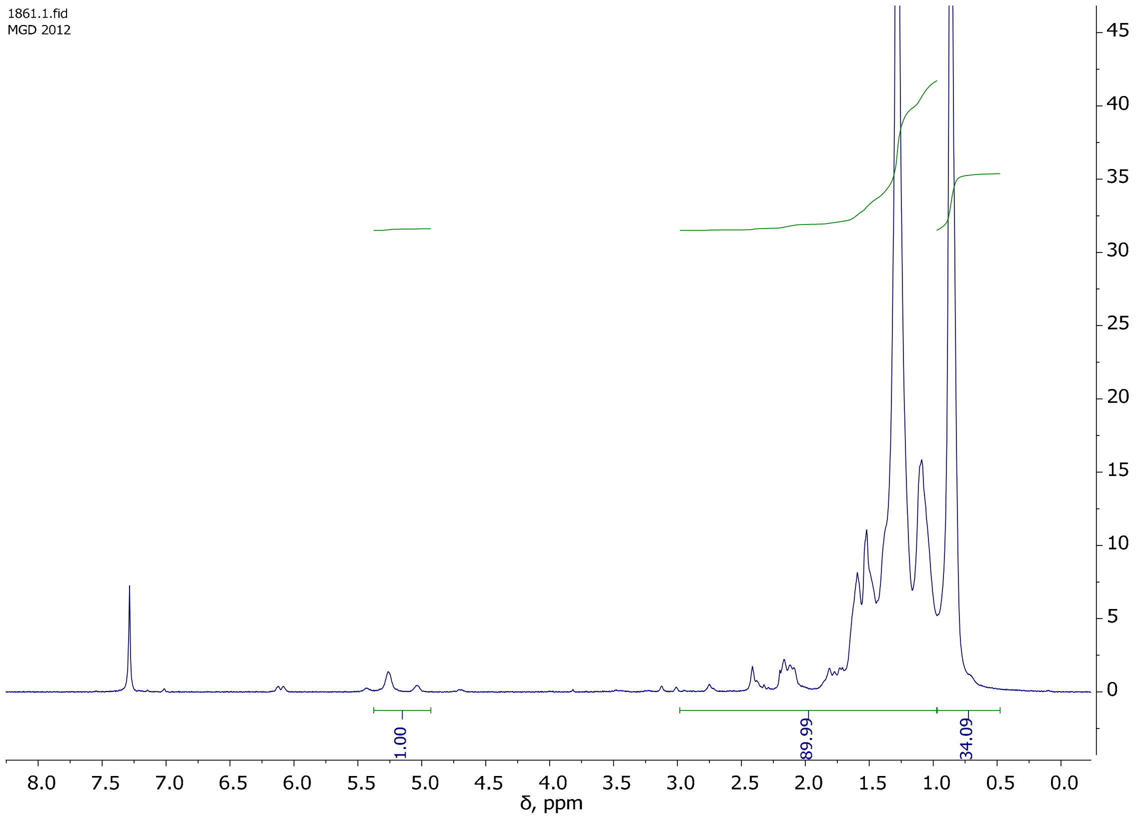

Figure S31. ^{1}H NMR spectrum of E/P/ 5-Vinyl-2-norbornene ter-copolymer (run 1, table 3).

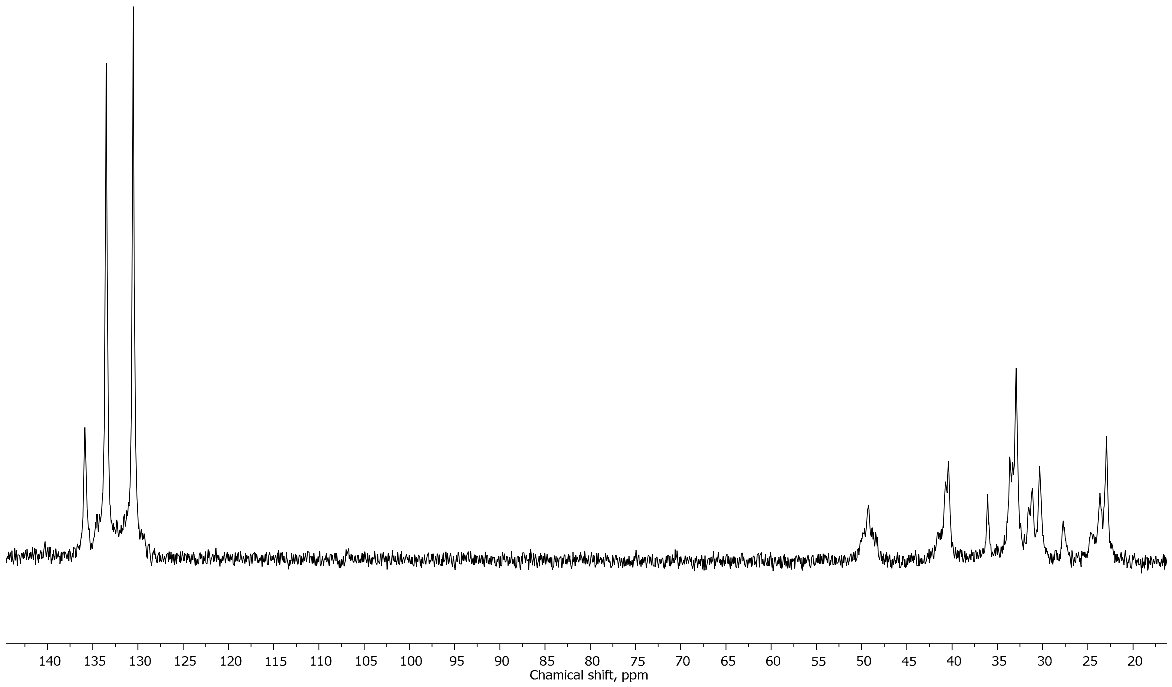

Figure S32. ^{13}C NMR spectrum of E/P/ 5-Vinyl-2-norbornene ter-copolymer (run 1, table 3).

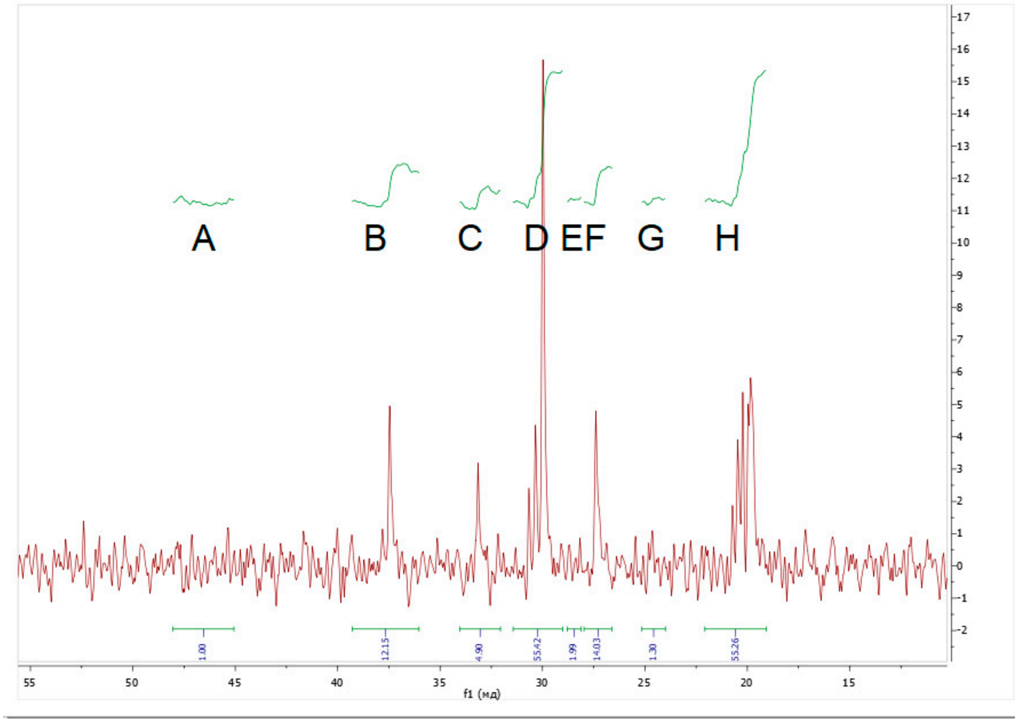

Figure S33. ^{13}C NMR spectrum of E/P/ 5-Vinyl-2-norbornene ter-copolymer (run 2, table 3).

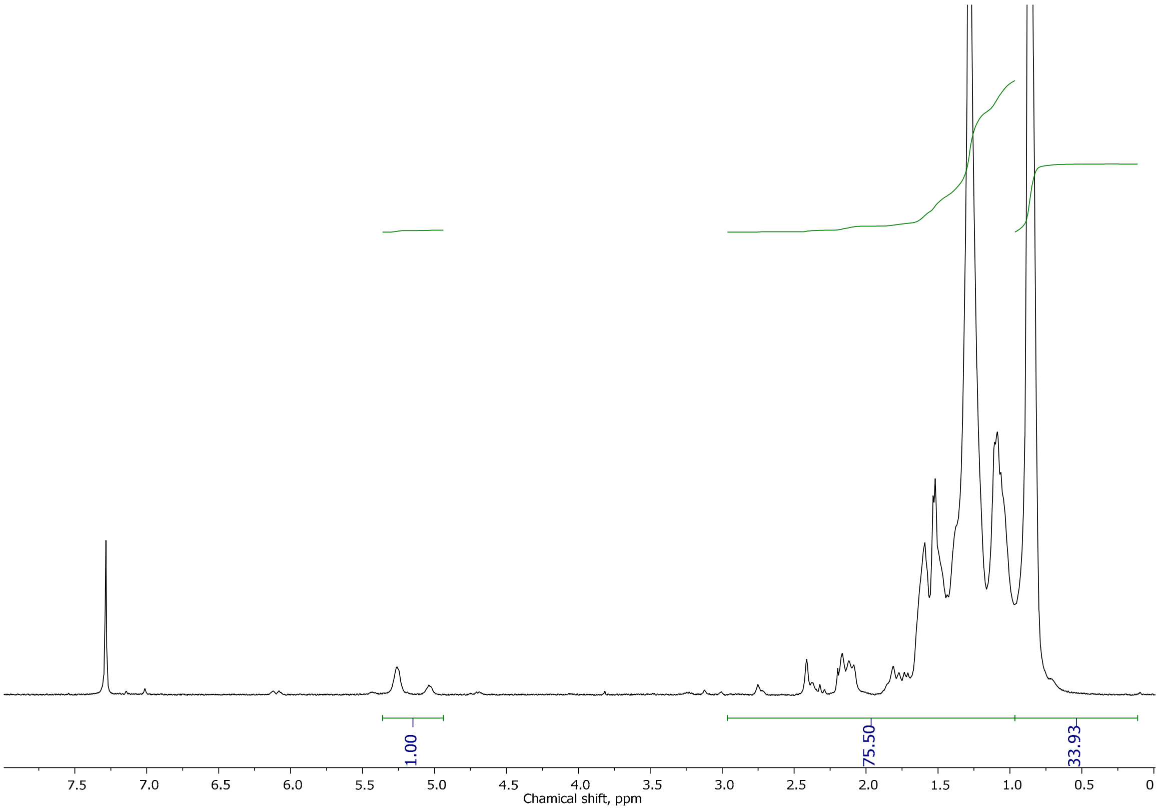

Figure S34. ^{1}H NMR spectrum of E/P/ 5-Vinyl-2-norbornene ter-copolymer (run 3, table 3).

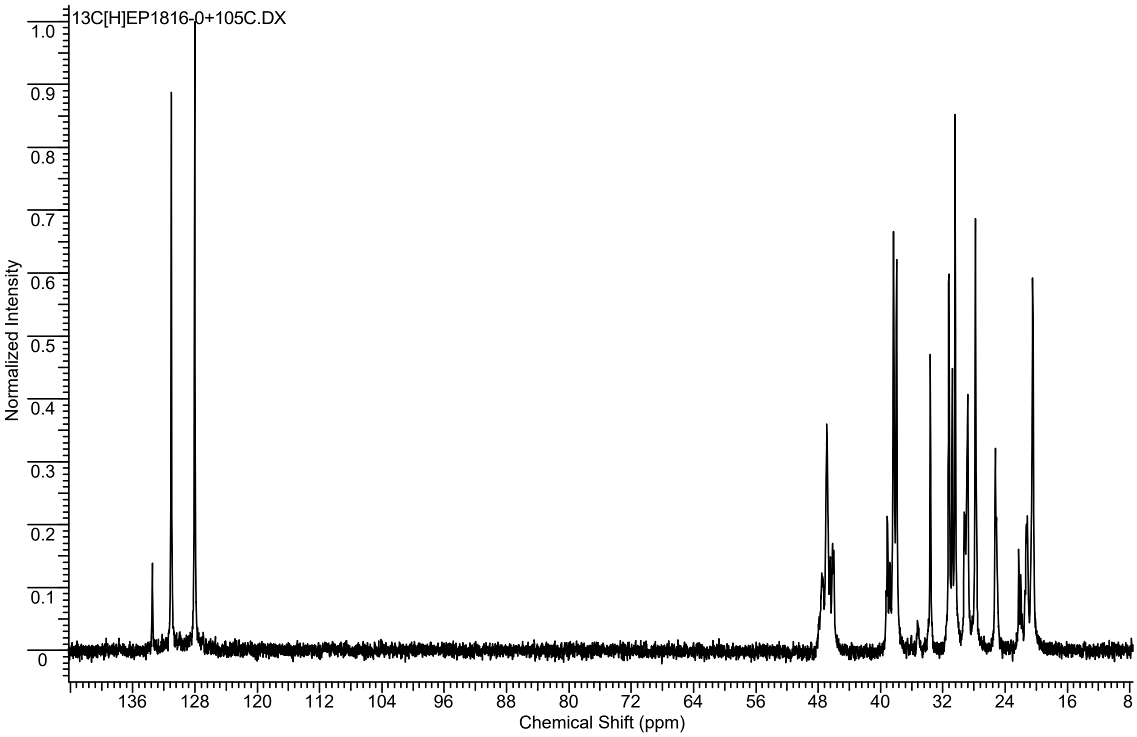

Figure S35. ^{13}C NMR spectrum of ter-copolymer (run 3, table 3).

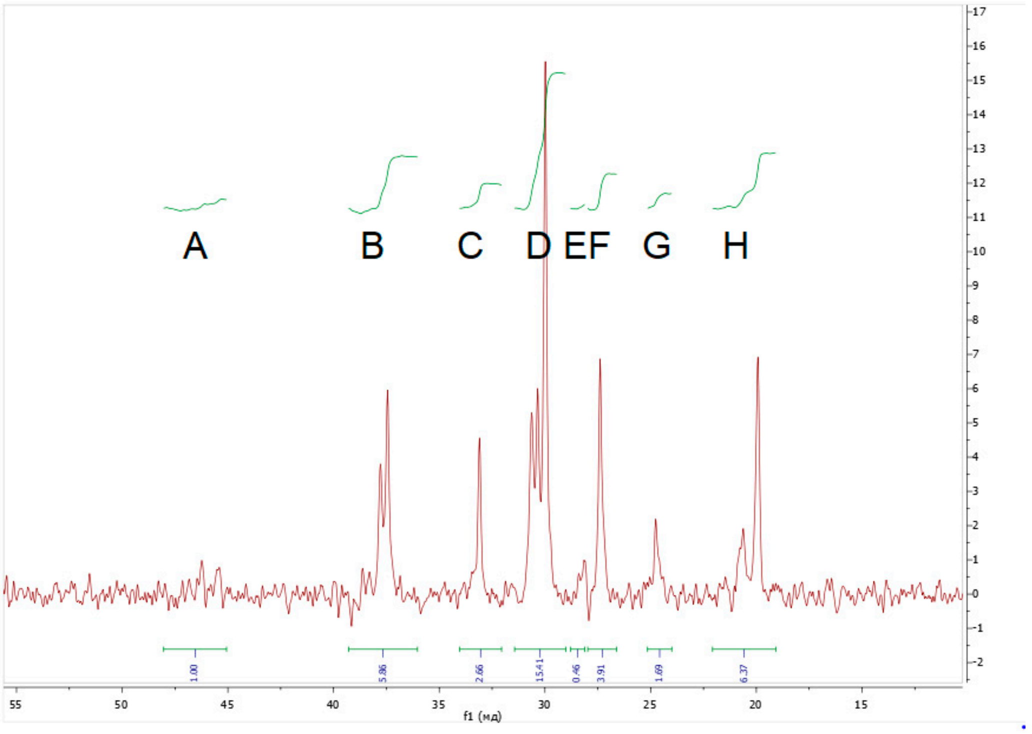

Figure S36. ^{13}C NMR spectrum of ter-copolymer (run 4, table 3).

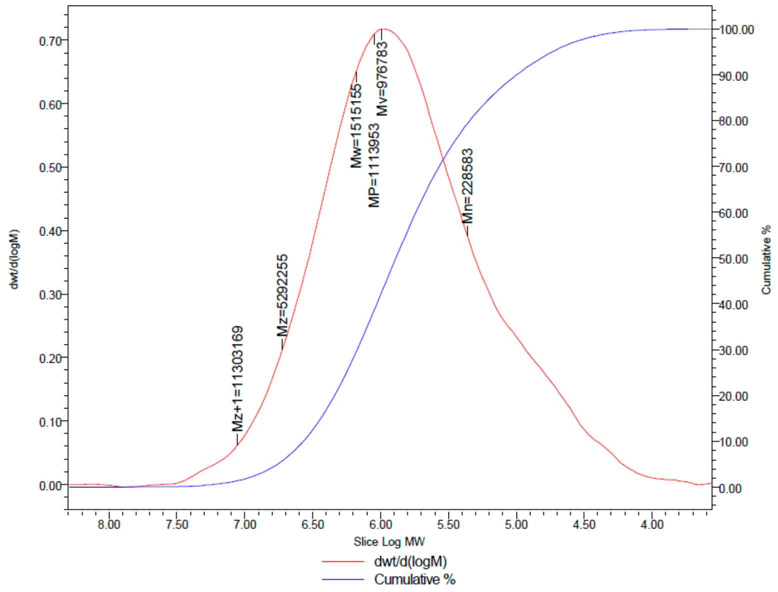

Figure S37. GPC curves of ethylene copolymers (table 3, entry 1).

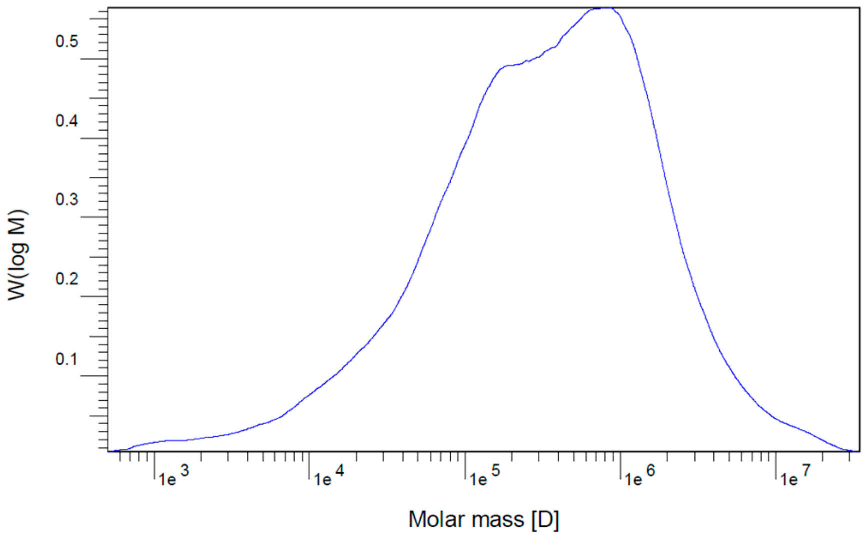

Figure S38. GPC curves of ethylene copolymers (table 3, entry 2)

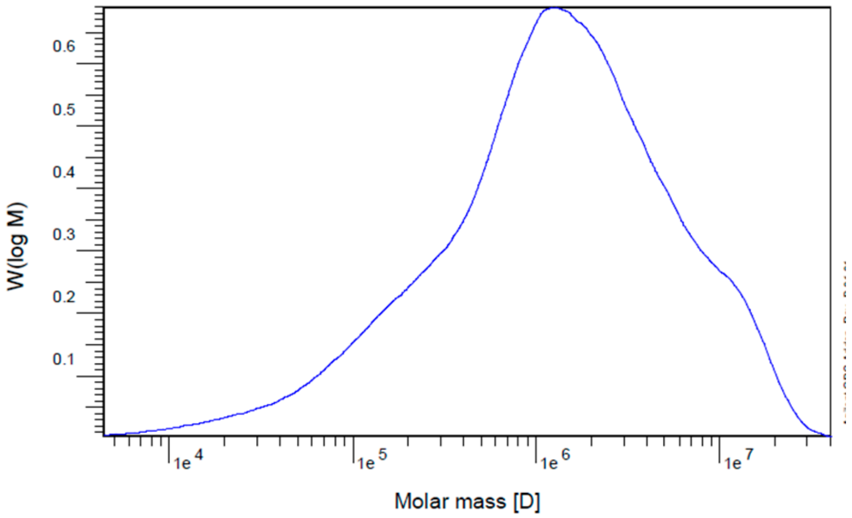

Figure S39. GPC curves of ethylene copolymers (table 3, entry 3)

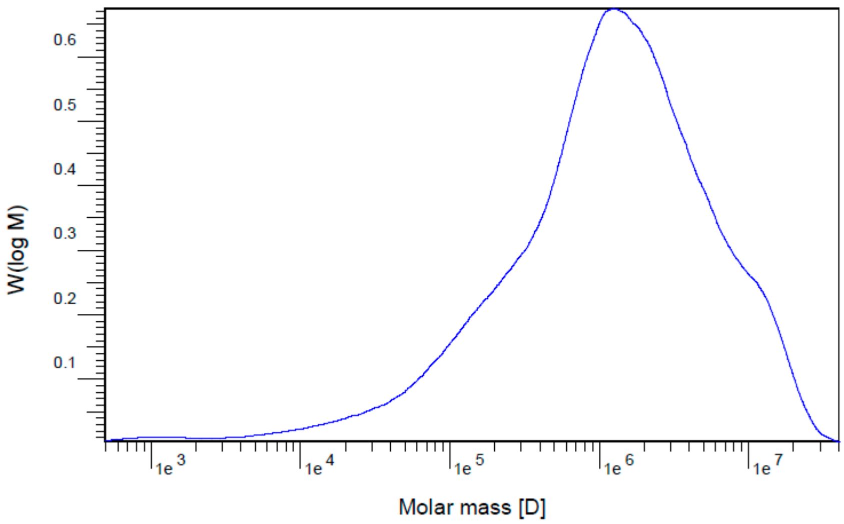

Figure S40. GPC curves of ethylene copolymers (table 3, entry 4)

Figures S1–S14: ^{1}H NMR and ^{13}C NMR spectrums of complex; Figures S15–S19: SEM images of the surface morphology of UHMWPE powders; Figures S20–30: DSC curves corresponding to UHMWPE; Figures S31–S36: ^{1}H NMR and ^{13}C NMR spectrum of ter-copolymer; Figures S37–40: GPC curves of ethylene copolymers; Table S1:Crystal data and structure refinement parameters for $(L1)_2Ti(OiPr)_2$, $(L1)_2Zr(OiPr)_2$, $(L2)_2Zr(OiPr)_2$, and $(L1)_2Zr(Cl)_2$; Table S2: Selected bond lengths (A) and angles (deg) for complexes $(L1)_2Ti(OiPr)_2$, $(L1)_2Zr(OiPr)_2$, $(L2)_2Zr(OiPr)_2$ and $(L1)_2Zr(Cl)_2$.
